# Social health protection to improve access to health care for people with disabilities

**DOI:** 10.2471/BLT.21.286685

**Published:** 2021-08-01

**Authors:** Lena Morgon Banks, Hannah Kuper, Tom Shakespeare

**Affiliations:** a London School of Hygiene & Tropical Medicine, Keppel Street, London, WC1E 7HT, England.

This year marks the tenth anniversary of the *World report on disability.*^1^ In this seminal publication of the World Health Organization and World Bank, evidenced-based estimates showed that 15% of the global population was living with disabilities. Also provided in the report were robust data on barriers to inclusion in key areas of life where people with disabilities are often left behind, and on strategies to redress them. However, the report barely mentioned social health protection, a relatively new policy tool at the time.

People with disabilities typically need more health care than the rest of the population because they require disability-related services and assistive devices on top of general health services.^1^^,^^2^ However, their access to care is hindered by informational, financial, physical and attitudinal barriers.^2^ Cost is a major barrier to accessing health care. People with disabilities have higher health costs but lower capacity to pay because they use more health services and are overrepresented among people living in poverty.^3^ Their health-related costs vary with the type and degree of impairment, age of onset and presence of comorbidities and secondary conditions.^4^^,^^5^ The *World report on disability* revealed that half of the people with disabilities in 51 surveyed countries could not afford needed health care and that, overall, people with disabilities were more likely to incur catastrophic health expenditures.^1^ They are also at risk of exclusion from universal health coverage (UHC) for lack of sufficient financial protection.^2^

Social health protection is critical to achieving sustainable development goal (SDG) 3 – to ensure a healthy life for all – and its target 3.8 – to achieve UHC.^6^ Social health protection schemes cover some or all of the costs of health services and products within a defined benefit package and are financed through taxes or individual contributions. Some schemes offer all citizens and residents automatic, mandatory or voluntary enrolment; others target vulnerable groups.

Social health protection programmes are increasingly being implemented in middle-income and even low-income countries.^6^^,^^7^ However, countries at all income levels must examine the extent to which programme design and delivery are inclusive of people with disabilities. First, programmes must review and address barriers to enrolment, including eligibility criteria that inadvertently exclude people with disabilities. For example, health insurance is commonly tied to formal sector employment. However, people with disabilities are more likely to be unemployed or to work in the informal sector.^1^^,^^8^ Even those who are eligible may not enrol due to access barriers (e.g. physically inaccessible application points, lack of information in alternative communication formats); stigmatization by programme staff; or poor awareness of available programmes.^9^^,^^10^

Second, programmes must assess whether covered health-care services and products have the range and quality to adequately meet the health needs of people with disabilities. Many existing programmes provide insufficient coverage for disability-related services and assistive devices or only cover services of lesser quality.^6^^,^^11^ This gap results in unmet health needs or in out-of-pocket payments for private care.^11^

Third, programmes must assess whether people with disabilities seeking health care are receiving sufficient financial protection. Even if fully covered for all their needed services and products, people with disabilities may still face impoverishment from frequent or high user fees and are more likely to incur indirect costs.^12^ For example, the paucity and centralization of disability-related health services in many countries, plus the lack of accessible public transportation, can make travel costs prohibitive for people with disabilities seeking health care.^2^^,^^6^

We now need further research to understand how social health protection affects people with disabilities and to learn how to design disability-inclusive programmes that will lead to success in attaining SDG 3 and UHC.
